# Multi‐omics analysis highlights *TM4SF4* ^+^ epithelial cells and the MMP14‐associated signaling program in lung adenocarcinoma pathogenesis and microenvironment remodeling

**DOI:** 10.1002/ccs3.70114

**Published:** 2026-11-13

**Authors:** Jingbo Li, Jiahua Liu, Guangxuan Liu, Kaiyu Wang, Qi Yan, Tianhao Sun, Shuo Chen, Kai Fu

**Affiliations:** ^1^ Department of Thoracic Surgery The First Affiliated Hospital of Harbin Medical University Harbin China; ^2^ Department of Anesthesiology The First Affiliated Hospital of Heilongjiang University of Chinese Medicine Harbin China

**Keywords:** cell communication, lung adenocarcinoma, *MMP14*, *TM4SF4*, tumor microenvironment

## Abstract

Lung adenocarcinoma (LUAD) progression is shaped by aberrant cell–cell and cell–matrix communication within a heterogeneous tumor microenvironment (TME). This study integrates single‐cell RNA sequencing (scRNA‐seq), genome‐wide association study, and spatial transcriptomics to identify epithelial subpopulations associated with this crosstalk. Analyzing GSE196303 scRNA‐seq data, we resolved 15 epithelial subpopulations. Using scPagwas, the *TM4SF4* ^+^ subpopulation showed the highest trait‐relevance score for LUAD genetic susceptibility (adjusted *p* < 0.05). BayesPrism deconvolution of TCGA‐LUAD data revealed *TM4SF4* ^+^ cell enrichment in tumors; high abundance was associated with poorer overall survival in the median‐cutoff analysis (hazard ratio = 1.43, 95% confidence interval = 1.07–1.92, *p* = 0.016) and with TME remodeling features (reduced immune/stromal scores and increased tumor purity). Weighted gene co‐expression network analysis and multi‐evidence intersection identified *MMP14* as a candidate hub gene. Spatial transcriptomics supported the heterogeneous distribution of *MMP14*, and functional enrichment linked *MMP14*‐associated transcriptional programs to ECM remodeling, migration, and Hedgehog/Notch pathways. Expression validation confirmed *MMP14* upregulation in LUAD cells and tissues. Collectively, *TM4SF4* ^+^ epithelial cells and the *MMP14*‐associated program may contribute to LUAD progression and TME remodeling, supporting them as candidate biomarkers and potential therapeutic targets for further validation.

## Graphical abstract

This study integrates single‐cell RNA sequencing, GWAS, and spatial transcriptomics to identify *TM4SF4* ^+^ epithelial cells as a LUAD‐associated subpopulation. The MMP14‐associated program links epithelial heterogeneity with tumor microenvironment remodeling and potential prognostic relevance.

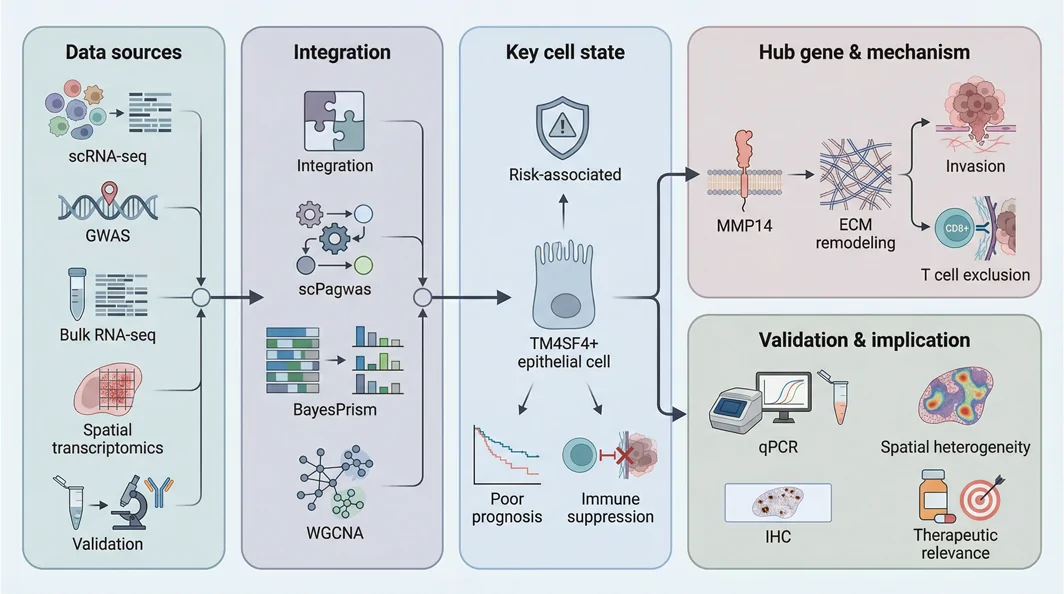

## 1 INTRODUCTION

Lung cancer remains one of the most lethal malignant tumors worldwide, with non‐small‐cell lung cancer (NSCLC) comprising the majority of cases. Among its subtypes, lung adenocarcinoma (LUAD) is the most prevalent.^1^, ^2^, ^3^ Global statistics from 2022 indicate that lung cancer continues to rank among the leading causes of new cases and mortality.^1^ Although targeted therapies and immune checkpoint inhibitors (ICIs) have improved outcomes for certain patients, significant variability in therapeutic efficacy, along with frequent recurrence and drug resistance, persists. The mechanisms underlying immune therapy tolerance and acquired resistance are complex and constitute major barriers to long‐term clinical benefits.^4^

The pronounced intratumoral heterogeneity in LUAD represents one of the primary sources of therapeutic uncertainty. This heterogeneity arises not only from the genomic evolution and phenotypic plasticity of tumor cells but also from the dynamic remodeling of the tumor microenvironment (TME).^5^, ^6^ Within the TME, immune cells, stromal cells, and the extracellular matrix (ECM) collectively influence immune evasion, invasion‐metastasis, and therapeutic response, while undergoing reconfiguration under treatment pressure.^7^, ^8^ Studies suggest that intratumoral heterogeneity can impact neoantigen presentation and immune recognition, thereby facilitating resistance to immunotherapy.^9^ Furthermore, the TME can promote the development of “cold tumors” and weaken therapeutic responses through mechanisms such as metabolic alterations, immune cell dysfunction, and physical barriers.^10^

To establish a more interpretable bridge between mechanisms and clinical applications, it is essential to identify “cell states” and driver networks that reflect disease processes. Single‐cell RNA sequencing (scRNA‐seq) has revealed the extensive heterogeneity of tumor cells and the TME in LUAD, as well as systematic associations between diverse microenvironmental patterns and tumor cell states.^11^ Spatial transcriptomics has further demonstrated that cellular plasticity and microenvironmental remodeling during tumor progression exhibit spatial compartmentalization and gradient characteristics.^12^, ^13^ On the other hand, genome‐wide association study (GWAS) has identified multiple risk loci associated with lung cancer/LUAD, but functional interpretations of these findings often require mapping back to specific cell types and states.^14^

Against this backdrop, tumor epithelial cell subpopulations, which directly embody core malignant phenotypes such as proliferation, invasion, and metastasis, have emerged as a focal point of interest. Transmembrane 4 superfamily member 4 (*TM4SF4*) has been implicated in adhesion and signal integration and may influence immune evasion. Investigations into *TM4SF4* suggest its potential therapeutic value and relevance to immune‐related regulation.^15^ Simultaneously, remodeling of the ECM modulates cell–matrix communication and impacts immune cell recruitment and function.^16^, ^17^ Matrix metalloproteinase 14 (*MMP14*), also known as MT1‐MMP, is recognized as a critical factor in ECM degradation and tumor invasion, and it also mediates intercellular and cell‐matrix signaling.^18^

Building upon the aforementioned context, this study aims to systematically investigate the biological significance and potential clinical value of key epithelial cell subpopulations in LUAD by examining tumor heterogeneity and the cellular ecosystem. Through a comprehensive understanding of tumor cell states, the study seeks to elucidate the mechanistic roles of epithelial cell heterogeneity in tumor initiation and progression and to explore its intrinsic connections with genetic background, microenvironmental regulation, and disease progression. Scientifically, this research is expected to advance LUAD studies from traditional molecular‐level analyses to an integrative perspective centered on cellular states, thereby enriching the theoretical framework of tumor heterogeneity. Clinically, identifying functionally critical cell subpopulations and their associated molecular characteristics may offer a novel theoretical foundation for risk stratification, prognosis evaluation, and the optimization of precision therapeutic strategies. Particularly in light of the marked interindividual variability in immunotherapy efficacy, a deeper understanding of interactions between epithelial cell subpopulations and the TME could yield significant insights into improving therapeutic response rates and developing innovative intervention strategies.

## 2 MATERIALS AND METHODS

### 2.1 Study design and overall workflow

This study used an integrated strategy of “single‐cell analysis—genetic association—cohort validation—mechanism exploration—experimental validation” to systematically identify critical epithelial cell subsets and their molecular hubs in LUAD. Furthermore, it evaluated their associations with prognosis, TME, somatic mutations, and potential drug responses. The specific workflow included: constructing cellular atlases through scRNA‐seq and analyzing epithelial subsets; integrating GWAS data to perform scPagwas for identifying LUAD susceptibility‐related key subsets; using BayesPrism deconvolution to estimate epithelial‐subset abundance (theta) in The Cancer Genome Atlas (TCGA)‐LUAD and conducting differential abundance, survival, and microenvironment analyses; comparing phenotypic differences by integrating somatic mutations and drug‐sensitivity predictions from oncoPredict; and finally, pinpointing candidate hub genes through weighted gene co‐expression network analysis (WGCNA) and multi‐evidence intersection, followed by mechanism and experimental validations using gene set enrichment analysis (GSEA), spatial transcriptomics, and reverse transcription quantitative polymerase chain reaction (RT‐qPCR)/immunohistochemistry (IHC). The primary endpoint was overall survival (OS); for stratified analyses, *TM4SF4* ^+^ abundance and candidate gene expression were categorized into high/low groups based on the median cutoff. The corresponding workflow diagram is illustrated in Supporting Information S1: Figure S1.

### 2.2 Data acquisition and sample inclusion/exclusion criteria

scRNA‐seq data are obtained from the Gene Expression Omnibus (GEO) dataset GSE196303 (https://www.ncbi.nlm.nih.gov/geo/), which includes three LUAD tumor samples and three matched adjacent non‐tumor samples. Downstream analyses use the author‐provided single‐cell expression matrices; raw count matrices serve as input for quality control and subsequent processing. Bulk transcriptomic data, somatic mutation profiles, and clinical follow‐up information are retrieved from the TCGA‐LUAD cohort, comprising 541 LUAD tumor tissues and 59 normal lung tissues. All available samples are included in the tumor–normal differential expression analysis. Survival analyses use OS as the primary endpoint. Cases with missing survival information or OS < 30 days are excluded; the final sample size for survival analyses is determined after filtering. Clinical variables are extracted and encoded from TCGA annotation files: age is modeled as a continuous variable (years at diagnosis), gender is coded as male/female, and pathological stage is categorized as Stages I–IV (and, when appropriate, collapsed into Stages I–II vs. III–IV). The T, N, and M categories are extracted from the corresponding fields and included as covariates in the regression adjustment when applicable. LUAD GWAS summary statistics are obtained from the IEU OpenGWAS database (https://gwas.mrcieu.ac.uk/), including 1002 cases and 462,008 controls. GWAS quality control excludes variants with minor allele frequency (MAF) < 0.05 and loci showing substantial deviation from Hardy–Weinberg equilibrium (HWE *p* < 1 × 10^−6^). Linkage disequilibrium (LD) processing is performed using LDlink, and variants are retained at the genome‐wide significance threshold (*p* < 5 × 10^−8^). Because LD thresholds are applied for different analytic purposes, this study uses *r* ^2^ > 0.8 to retrieve strong‐LD proxy variants and retains those that remain significantly associated with the corresponding lead single‐nucleotide polymorphism (SNPs). Spatial transcriptomics validation is performed using two public datasets, GSM5702474 (LUAD) and GSM5388415 (normal distal lung), to characterize the spatial distribution of *MMP14* expression in lung cancer tissue.

### 2.3 GWAS data processing and quality control

LUAD GWAS summary statistics are retrieved from the IEU OpenGWAS database (1002 cases and 462,008 controls). Quality control is applied to SNP‐level results by excluding variants with MAF < 0.05 and removing loci that show marked deviation from HWE (*p* < 1 × 10^−6^). Genome‐wide significant association signals are then defined using the conventional threshold (*p* < 5 × 10^−8^). To avoid conflating strong LD with statistical independence, a two‐step LD handling strategy is implemented. First, LD clumping is performed to obtain a set of approximately independent lead SNPs. Second, for each lead SNP, proxy variants in strong LD are retrieved for downstream mapping and weighting analyses. Specifically, clumping is conducted using a population‐matched reference panel consistent with the GWAS ancestry (EUR by default; EAS for East Asian GWAS), with a ± 250‐kb window (or an equivalent 1‐Mb span, depending on LDlink parameterization) and an independence threshold of *r* ^2^ < 0.1 (*r* ^2^ < 0.01 in sensitivity analyses). After defining the lead SNP set, LDlink is used to compute LD and identify proxy variants, applying a strong‐LD criterion of *r* ^2^ > 0.8. LDlink queries are performed using the same reference population (EUR or EAS) and consistent window settings. Variants that are in strong LD with lead SNPs and pass the significance filter are retained to construct input datasets for downstream scPagwas analyses.

### 2.4 scRNA‐seq data processing and cell annotation

The GSE196303 dataset is preprocessed and analyzed in R using Seurat (v4.0). Cells are first subjected to quality control to remove low‐quality profiles, excluding cells with fewer than 200 detected genes (nFeature_RNA < 200) or with a high mitochondrial transcript proportion (percent.mt > 15%). In addition, cells with extremely high unique molecular identifier counts are filtered to reduce potential doublet contamination, with thresholds determined from the upper tail of the sample‐specific nCount_RNA distribution. Data are then normalized and feature‐selected. Log‐normalization is performed using NormalizeData, and 2000 highly variable genes are identified with FindVariableFeatures. The expression matrix is scaled with ScaleData, regressing out technical covariates, and then subjected to principal component analysis (PCA) to derive low‐dimensional representations for downstream analyses. To mitigate batch effects across samples, Harmony is applied in PCA space, and Harmony embeddings are used for clustering and visualization. A shared nearest‐neighbor graph is constructed using FindNeighbors, followed by graph‐based clustering with FindClusters. Multiple resolution parameters are evaluated using a clustree‐based numerical resolution grid (0.01, 0.05, 0.1, 0.2, 0.3, 0.5, 0.8, 1, 1.2, 1.5, 2, 2.5, and 3), and the final whole‐atlas clustering resolution is set to 1 based on cluster‐stability assessment and dendrogram continuity. Dimensionality reduction for visualization is performed using uniform manifold approximation and projection (UMAP) or t‐distributed stochastic neighbor embedding (t‐SNE) (RunUMAP/RunTSNE). Cell‐type annotation is determined by integrating canonical marker gene expression with differential expression results. Marker genes are identified using FindMarkers and cross‐validated against the CellMarker 2.0 database and relevant literature, yielding annotations for major cell types and epithelial subpopulations.

### 2.5 Epithelial cell extraction and secondary clustering

After performing clustering and cell‐type annotation at the whole‐dataset level, cells annotated as epithelial are extracted for secondary analysis. Epithelial cell selection is based on the initial annotations and further verified by the expression of canonical epithelial markers, including EPCAM and multiple keratin genes, to ensure a well‐defined epithelial lineage. The epithelial subset is then reprocessed using a standard Seurat workflow, including normalization, identification of highly variable genes, data scaling, and PCA. A nearest‐neighbor graph is constructed in the integrated low‐dimensional space, followed by secondary clustering. The number of principal components (dims) and the clustering resolution are determined based on the elbow plot and cluster‐stability assessment; the whole‐atlas resolution grid and final resolution are reported in Supporting Information S1: Figure S2, and epithelial‐subset clustering is then performed in the integrated low‐dimensional space. UMAP or t‐SNE is used for visualization, enabling the identification of 15 epithelial subpopulations across tumor and adjacent tissues. Marker genes for each epithelial subpopulation are identified using FindMarkers. Differentially expressed genes (DEGs) are filtered using predefined thresholds for the proportion of expressing cells and log fold change (FC) and then ranked by statistical significance and effect size. Top‐ranked representative markers are used for subpopulation annotation and downstream functional analyses.

### 2.6 scPagwas genetic association analysis

The scPagwas package (v1.2.0) in R is used to evaluate associations between epithelial cell subpopulations and LUAD genetic susceptibility. Briefly, quality‐controlled GWAS summary statistics are used as input, with SNP effect sizes representing the genetic signal. In parallel, pathway activity scores are computed for individual cells and aggregated at the subpopulation level to serve as phenotypic features. Pathway gene sets are obtained from public annotation resources and incorporated into a pathway‐based multigene regression framework. Specifically, GWAS SNP effect sizes are treated as predictors and single‐cell‐inferred pathway activities as responses; weighted linear regression is performed with the absolute SNP effect size used as the weighting factor to quantify the strength of association between genetic variation and pathway activity. For each epithelial subpopulation, *p*‐values are computed for pathway–trait associations. Multiple testing across all subpopulation–pathway combinations is controlled using Bonferroni correction, and an adjusted *p* < 0.05 is considered statistically significant. For significantly associated pathways, genes within each pathway are prioritized by the product of “GWAS effect size × scRNA‐seq expression level,” and the top 10% of ranked genes constitute the core candidate gene set for that pathway. To summarize genetic relevance at the subpopulation level, a trait‐relevance score (TRS) is calculated by integrating association effect sizes and corrected significance across pathways for each subpopulation. The robustness of TRS differences is assessed using bootstrap resampling, with *p* < 0.05 indicating statistical significance. Finally, epithelial subpopulations within the top 5% of TRS rankings are defined as key subpopulations most strongly associated with LUAD genetic susceptibility and are carried forward for cohort‐level validation and mechanistic analyses.

### 2.7 BayesPrism

To quantify epithelial cell subpopulation composition in TCGA‐LUAD bulk RNA sequencing (bulk RNA‐seq) data, BayesPrism (R package v1.1.0) is used for single‐cell reference‐based deconvolution. The reference expression profile is constructed from 15 epithelial subpopulations defined by secondary clustering of the scRNA‐seq dataset. For each subpopulation, cell‐level count data are aggregated to generate a subpopulation‐specific reference matrix. Reference genes are selected to maximize subpopulation specificity, prioritizing features with strong discriminative power and sufficient expression (area under the receiver operating characteristic curve [AUROC] > 0.8 and mean expression >1). Genes selected across subpopulations are combined to form the final reference gene set. For deconvolution input, raw count matrices from TCGA‐LUAD are used, and only genes overlapping with the reference profile are retained for model fitting.

BayesPrism is fitted using a Dirichlet‐multinomial likelihood to accommodate count‐based expression data, with an additional gene‐length correction term to mitigate transcript length bias. Markov chain Monte Carlo sampling is run for 10,000 iterations, with the first 2000 iterations discarded as burn‐in. Convergence is assessed by examining the stability of key parameter/proportion trace plots and the consistency of posterior distributions. Cell subpopulation abundance is summarized as theta, defined as the posterior median of the inferred proportion of each epithelial subpopulation within each bulk sample. Theta serves as the unified input for all downstream abundance‐related analyses, including differential abundance testing, survival modeling, microenvironment characterization, and mutation/drug association analyses. When multiple tumor samples are available for the same patient, a patient‐level aggregation strategy is applied to derive a single theta value (by default, the median across samples to reduce sensitivity to outliers). If a patient contributes only one tumor sample, the corresponding theta is directly used for patient‐level stratification and regression analyses.

### 2.8 Differential expression analysis of bulk RNA‐seq data

Bulk RNA‐seq expression data from the TCGA‐LUAD cohort are analyzed using raw count matrices as input for preprocessing and differential expression analysis. Analyses are performed in R with the limma package (v3.40.6), incorporating the voom transformation to model the mean–variance relationship of count data before linear modeling. Differential expression statistics are obtained using the empirical Bayes framework implemented in limma to improve variance estimation and statistical stability. Comparisons are conducted between tumor and normal tissue samples. Genes meeting the criteria of |log_2_FC| > 1 and false discovery rate (FDR) < 0.05 after Benjamini–Hochberg (BH) multiple testing correction are defined as DEGs. Visualization of the results, including volcano plots and heatmaps, is generated using R packages such as ggplot2, with the BH‐adjusted FDR applied consistently across analyses.

### 2.9 Prognostic analysis and cutoff sensitivity assessment

OS is used as the primary endpoint in the TCGA‐LUAD cohort, with follow‐up time and vital status obtained from the clinical annotation files. Samples lacking survival information or with OS < 30 days are excluded before analysis. Patients are stratified according to BayesPrism‐derived epithelial subpopulation abundance (theta; posterior median) or candidate gene expression, and Kaplan–Meier (KM) survival curves are generated. Between‐group differences are evaluated using the log‐rank test. In parallel, Cox proportional hazards models are fitted for univariable and multivariable analyses, reporting hazard ratios (HRs), 95% confidence intervals (CIs), and *p*‐values. The default stratification uses the median value as the cutoff to define high and low groups. To examine the robustness of results to cutoff selection, cutoff sensitivity analyses are additionally performed, including a tertile‐based strategy (upper one third vs. lower one third) and an optimal cutpoint approach. For the latter, the surv_cutpoint function in the survminer package is used to identify the cutpoint that minimizes the log‐rank *p*‐value within a prespecified search range, while enforcing a minimum group proportion to avoid overly small strata. Consistency in effect direction and statistical significance across strategies is assessed. Multivariable Cox models adjust for clinical covariates, including age, gender, and pathological stage, to evaluate independent prognostic value. When appropriate, the proportional hazards assumption is tested to confirm model validity.

### 2.10 Immune microenvironment assessment and association modeling

To quantify the relationship between *TM4SF4* ^+^ epithelial cell abundance and the immune/stromal composition of the TME, the ESTIMATE algorithm is applied to the TCGA bulk RNA‐seq expression matrix to derive the stromal score, immune score, and ESTIMATE score and to infer tumor purity. For group‐based comparisons, patients are stratified into *TM4SF4* ^+^ high‐ and low‐abundance groups using the median abundance (theta) as the cutoff. Differences in each score are evaluated using a Student's *t*‐test or the Mann–Whitney *U*‐test, as appropriate based on distributional assumptions, and multiple testing across endpoints is controlled with the BH FDR procedure. To reduce dependence on an arbitrary cutoff, *TM4SF4* ^+^ abundance is additionally analyzed as a continuous variable. Associations with stromal score, immune score, ESTIMATE score, and tumor purity are quantified using Spearman correlation and/or linear regression, with correlation coefficients or regression estimates reported alongside *p*‐values. To account for potential confounding, adjusted regression models include pathological stage; a further model additionally incorporates tumor purity to evaluate whether *TM4SF4* ^+^ abundance is independently associated with immune and stromal scores.

### 2.11 WGCNA and hub gene prioritization strategy

A weighted gene co‐expression network is constructed from the bulk RNA‐seq expression matrix of TCGA‐LUAD tumor samples using the WGCNA package in R. Before network construction, genes are filtered to remove lowly expressed features, and a high‐variance gene set is retained for downstream analysis. The soft‐thresholding power (*β*) is selected using pickSoftThreshold to approximate scale‐free topology. An adjacency matrix is then computed and transformed into a topological overlap matrix, and co‐expression modules are identified via dynamic tree cutting. Module eigengenes are calculated and correlated with the *TM4SF4* ^+^ epithelial cell score/abundance. Multiple testing is controlled using the BH procedure. Modules meeting |*r*| > 0.3 and FDR < 0.05 are defined as significantly associated and carried forward for hub gene screening. To prioritize *TM4SF4* ^+^‐linked hub candidates, genes from the significant module(s) (e.g., MEtan) are intersected with tumor–normal DEGs in TCGA and marker genes of *TM4SF4* ^+^ epithelial cells identified from scRNA‐seq. The resulting intersected gene set is used for subsequent validation and functional analyses.

### 2.12 Somatic mutation profiling and differential mutation analysis

Somatic mutation data for TCGA‐LUAD are obtained in mutation annotation format (sourced from TCGA GDC and/or the MC3 harmonized mutation annotation format, with sample identifiers consistently matched to the corresponding clinical IDs). All analyses are conducted in R using the maftools package. Briefly, the mutation annotation format file is imported with read.maf to generate a mutation annotation format object. Tumor mutation burden, variant classifications, and recurrently mutated genes are summarized using functions such as getSampleSummary and getGeneSummary, and the mutational landscape and distribution of top mutated genes are visualized with oncoplot. To identify differential mutation patterns between *TM4SF4* ^+^ epithelial cell high‐ and low‐abundance groups, a 2 × 2 contingency table is constructed for each gene (mutant vs. wild type in the high‐abundance group vs. mutant vs. wild type in the low‐abundance group). Fisher's exact test is used to evaluate differences in mutation frequency, and odds ratios (ORs) with 95% CIs are reported. Genome‐wide multiple testing is controlled using the BH procedure for FDR adjustment. The genome‐wide summary plot in Figure 4D displays per‐gene ORs and 95% CIs for the low‐abundance group relative to the high‐abundance group, with significance annotations based on unadjusted Fisher's exact test *p*‐values; BH‐adjusted FDRs are provided in Supporting Information S2: Table S2.

### 2.13 Drug‐sensitivity prediction and comparative analysis

Drug response prediction is performed using bulk RNA‐seq expression profiles from the TCGA‐LUAD cohort and the oncoPredict R package. Model training is based on pharmacogenomic screening data from the Genomics of Drug Sensitivity in Cancer database (specifying either the GDSC1 or GDSC2 release). The calcPhenotype function is applied to project TCGA expression profiles into the drug response space, generating predicted response values for each sample‐drug pair. The output metric corresponds to the model‐derived estimate (typically predicted half maximal inhibitory concentration or a relative sensitivity score; if the drug‐sensitivity score is used, this is explicitly specified and consistently reported). Predicted drug responses are compared between *TM4SF4* ^+^ high‐ and low‐abundance groups for each compound. The Mann–Whitney *U*‐test is used by default, and *p*‐values across all tested drugs are adjusted using the BH procedure to control the FDR. Candidate agents are prioritized based on the effect size, defined as the difference in median predicted response between groups (Δmedian = high − low). After FDR filtering, the top 10 drugs with the largest positive Δmedian values are designated as relatively sensitive in the *TM4SF4* ^+^‐high group, whereas the top 10 with the most negative Δmedian values are classified as relatively resistant and are presented accordingly.

### 2.14 GSEA functional enrichment analysis

To elucidate the biological functions associated with *MMP14*‐related transcriptional programs, TCGA‐LUAD tumor samples are stratified into *MMP14*‐high and *MMP14*‐low groups using the median expression value as the cutoff. Differential expression analysis is then performed, and a pre‐ranked gene list is generated for GSEA. Genes are ranked by the test statistic derived from the differential analysis (preferentially the *t*‐value or an equivalent statistic; log_2_FC is used when appropriate) and used as input for enrichment testing. GSEA is conducted in R using fgsea (or clusterProfiler), with 10,000 permutations to ensure robust significance estimation. Enrichment analyses are performed separately for Kyoto Encyclopedia of Genes and Genomes (KEGG) and Reactome pathway collections. Gene sets are obtained from the curated Molecular Signatures Database (MSigDB) annotations for KEGG and Reactome. Gene identifiers are harmonized before analysis to ensure consistent mapping to the expression matrix. Enrichment results are summarized using the normalized enrichment score (NES) to indicate the direction and magnitude of enrichment. Statistical significance is assessed using FDR‐adjusted *q*‐values, with FDR *q* < 0.05 defined as the threshold for significant enrichment. For significantly enriched pathways, leading‐edge (core enrichment) genes are extracted for downstream mechanistic interpretation and visualization.

### 2.15 Spatial transcriptomics

Spatial validation of *MMP14* expression is conducted using two publicly available spatial transcriptomics sections, GSM5702474 and GSM5388415, obtained from the GEO. Data retrieval and visualization are performed on the STOmicsDB platform. Spatial feature plots are generated using the platform‐provided log‐normalized expression values. Color intensity (light to dark) represents increasing expression levels, enabling assessment of the intratumoral spatial distribution and heterogeneity of *MMP14*.

### 2.16 Cell culture

The human LUAD cell line A549 (RRID: CVCL_0023, male, lung, derived from adenocarcinoma) and the normal bronchial epithelial cell line BEAS‐2B (RRID: CVCL_0168, male, lung, normal bronchial epithelium immortalized with Ad12‐SV40) were obtained from the American Type Culture Collection (ATCC) in March 2023. Both cell lines were authenticated by the supplier using short tandem repeat profiling and were tested negative for mycoplasma contamination (last tested in February 2026 using a MycoAlert Mycoplasma Detection Kit, Lonza, Basel, Switzerland). Cells were cultured according to the supplier's instructions in RPMI‐1640 medium (Gibco) supplemented with 10% fetal bovine serum (FBS, Gibco). Cells were maintained at 37°C in a humidified incubator with 5% CO_2_, and the culture medium was replaced every 2–3 days. Cells in the logarithmic growth phase were harvested for subsequent experiments. To the best of our knowledge, neither cell line has been previously reported as misidentified or contaminated in the literature (based on the International Cell Line Authentication Committee database).

### 2.17 RT‐qPCR

Cells are washed twice with prechilled phosphate‐buffered saline, and total RNA is extracted using TRIzol reagent (Invitrogen) and stored at −80°C. Complementary DNA (cDNA) is synthesized by reverse transcription using the PrimeScript™ RT Reagent Kit (Takara) and stored at −20°C. *MMP14* primers are commercially synthesized with the following sequences: forward, 5′‐GGATACCCAATGCCCATTGGCCA‐3′; reverse, 5′‐CCTCGGTGCACCATGTTTGGC‐3′. RT‐qPCR is performed with three independent biological replicates (*n* = 3), and each biological replicate is analyzed in triplicate technical wells. A melt‐curve analysis is conducted after amplification to confirm product specificity. Relative expression is calculated using the 2^−ΔΔCt^ method, with BEAS‐2B serving as the reference for normalization. Group comparisons are performed using a two‐sided Student's *t*‐test, with statistical significance defined as *p* < 0.05.

### 2.18 Hematoxylin and eosin (H&E) staining and IHC

Formalin‐fixed paraffin‐embedded (FFPE) specimens were obtained from surgically resected LUAD tissues and matched adjacent non‐tumor tissues archived at The First Affiliated Hospital of Harbin Medical University. A total of six paired samples were included for histopathological evaluation. The study was approved by the Clinical Ethics Committee of The First Affiliated Hospital of Harbin Medical University, and written informed consent was obtained from all participants in accordance with the principles of the Declaration of Helsinki. For H&E staining, tissues were fixed in 4% neutral buffered formalin, dehydrated, cleared, and embedded in paraffin. Sections (4–5 μm) were deparaffinized, rehydrated, and processed following standard procedures, including hematoxylin staining, differentiation, bluing, eosin counterstaining, dehydration, clearing, and mounting. Representative microscopic fields were captured using a light microscope. For IHC, FFPE sections (3–5 μm) were deparaffinized and rehydrated, followed by antigen retrieval in citrate buffer. Endogenous peroxidase activity was blocked with hydrogen peroxide. Sections were incubated overnight at 4°C with a primary antibody against MMP14 (Proteintech, 14552‐1‐AP, 1:1000), followed by incubation with a horseradish peroxidase (HRP)‐conjugated secondary antibody at room temperature for 1 h. Signal detection was performed using DAB chromogen, followed by hematoxylin counterstaining. After dehydration and mounting, images were acquired under an Olympus BX53 microscope. MMP14 protein expression was semiquantitatively assessed using the H‐score method. Staining intensity was graded on a scale of 0–3 and integrated into a composite score ranging from 0 to 300 (H‐score = *Σ*[Pi × i], where Pi represents the percentage of positive cells at each intensity level i). Specific brown staining localized predominantly to the cell membrane and/or cytoplasm of tumor cells was considered positive. Scoring was independently performed by at least two evaluators blinded to group allocation; discrepancies were resolved through joint review and consensus. ImageJ software was additionally used for assisted quantitative analysis to enhance scoring consistency.

### 2.19 Statistical analysis

All statistical analyses are performed in R. For comparisons between two groups, normality is assessed first. When data are approximately normally distributed and satisfy homoscedasticity, a two‐sided Student's *t*‐test is applied; otherwise, the nonparametric Mann–Whitney *U*‐test is used. Comparisons among three or more groups are conducted using the Kruskal–Wallis test, followed by post hoc pairwise comparisons when appropriate. For analyses involving multiple endpoints or genome‐/drug‐wide testing, *p*‐values are adjusted for multiple comparisons using the BH procedure, with FDR <0.05 as the default significance threshold. Correlations are evaluated using Spearman's rank correlation, and both the correlation coefficient and *p*‐value are reported. Survival analyses use OS as the endpoint. KM curves are generated and compared with the log‐rank test, and Cox proportional hazards models are fitted for univariable and multivariable analyses, reporting HRs, 95% CIs, and *p*‐values. Statistical significance is defined as *p* < 0.05 or FDR <0.05. Significance annotations are as follows: *p < 0.05, **p < 0.01, ***p < 0.001, ****p < 0.0001, and ns (not significant).

## 3 RESULTS

### 3.1 Identification of core epithelial cell subpopulations in LUAD using scRNA‐Seq and scPagwas analysis

To systematically characterize the cellular composition and epithelial cell heterogeneity of LUAD at single‐cell resolution, we performed quality control and downstream analysis on publicly available scRNA‐seq datasets. First, we assessed the number of genes detected per cell (nFeature_RNA), the transcript count (nCount_RNA), the proportion of mitochondrial genes (percent.mt), and the proportion of ribosomal genes (percent.rb). Low‐quality cells were filtered out based on predefined criteria (Figure 1A). Clustering resolution was determined and evaluated for stability using the dendrogram/stability information now provided in Supporting Information S1: Figure S2. A total of 22 cell clusters were identified in LUAD and adjacent normal tissues (Figure 1B), which were annotated into seven major cell types based on classical marker genes: T cells, macrophages, natural killer cells, monocytes, epithelial cells, endothelial cells, and smooth muscle cells (Figure 1C).

**FIGURE 1:**
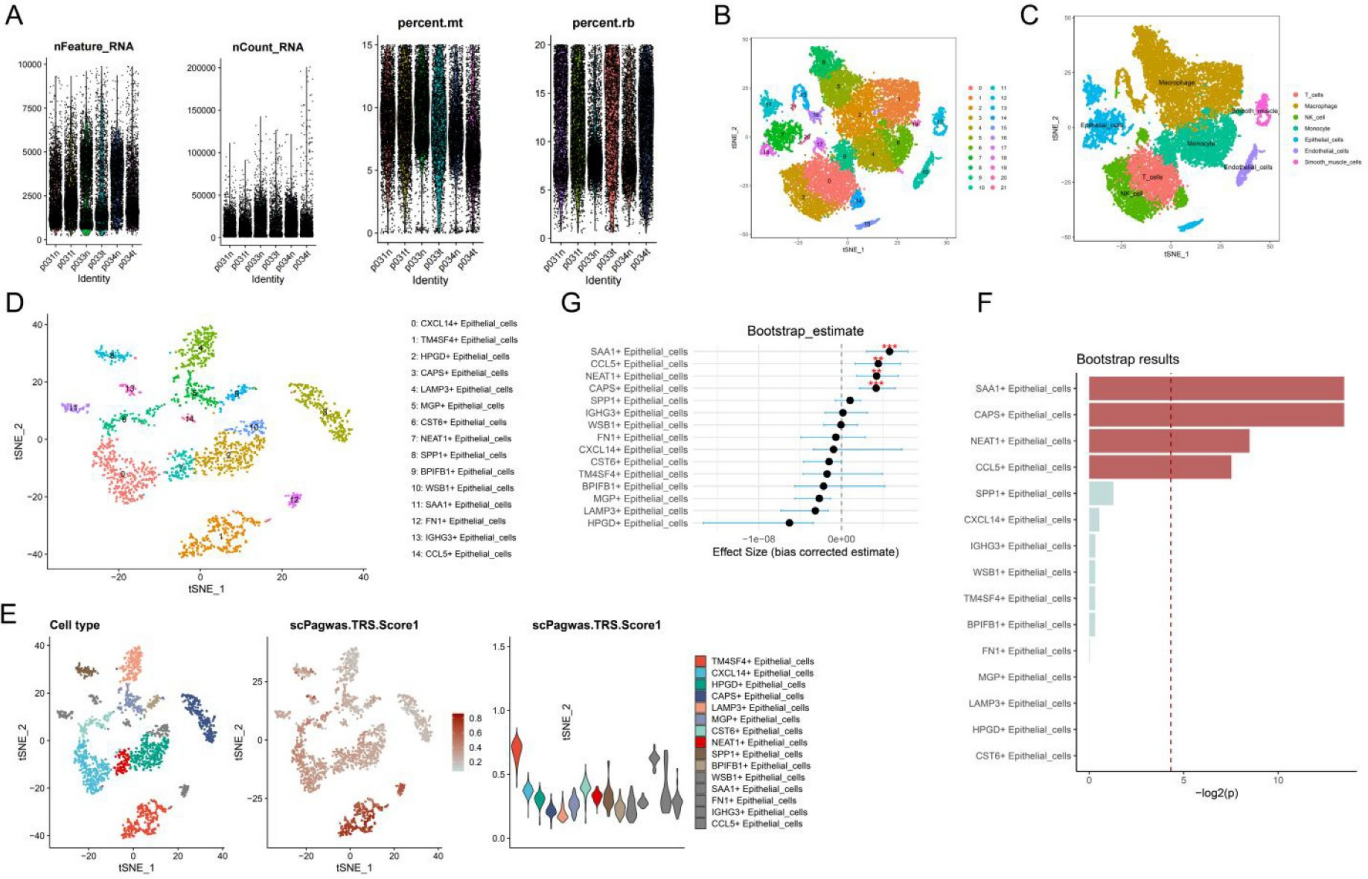
scRNA‐seq atlas and scPagwas identify *TM4SF4* ^+^ epithelial cells as a core subpopulation in LUAD. (A) Distributions of scRNA‐seq quality‐control metrics, including nFeature_RNA, nCount_RNA, percent. mt, and percent.rb; (B) t‐SNE/UMAP embedding of all cells showing 22 clusters; (C) annotation of seven major cell types based on marker gene expression; (D) t‐SNE embedding of epithelial cells after subset extraction and secondary clustering, resolving 15 epithelial subpopulations; (E) scPagwas TRS analysis in epithelial cells, showing the t‐SNE distribution of epithelial subpopulations, a TRS feature map in the embedding space (scPagwas.TRS.Score1), and violin plots of TRS across epithelial subpopulations; (F) significance testing based on bootstrap resampling, presented as −log2(p); and (G) effect size estimates and confidence intervals for LUAD risk across epithelial subpopulations. LUAD, lung adenocarcinoma; TRS, trait‐relevance score; t‐SNE, t‐distributed stochastic neighbor embedding.

Considering that tumorigenesis and genetic susceptibility signals are often enriched in epithelial cell populations, we further isolated epithelial cells for secondary clustering, identifying 15 epithelial cell subpopulations (Figure 1D). The representative marker gene expression patterns of each subpopulation, illustrated in the heatmap, revealed significant transcriptional heterogeneity within epithelial cells (Supporting Information S1: Figure S3). On this basis, LUAD GWAS summary statistics were integrated and analyzed using scPagwas to calculate TRS for each epithelial cell subpopulation. The results showed that the *TM4SF4* ^+^ epithelial cell subpopulation exhibited the highest TRS, with the TRS signals showing a concentrated spatial distribution, indicating that this subpopulation is most strongly associated with LUAD genetic risk (Figure 1E). Bootstrap resampling results and effect‐size estimates for the epithelial subpopulations are presented in Figure 1F,G. The top marker genes for each epithelial cluster are provided in Supporting Information S2: Table S1.

### 3.2 *TM4SF4* ^+^ epithelial cells are significantly enriched in TCGA‐LUAD and predict poor prognosis

To validate the reproducibility and clinical significance of epithelial cell subpopulations identified by single‐cell analysis in an independent large cohort, bulk RNA‐seq analysis and deconvolution inference were performed in the TCGA‐LUAD cohort. Differential expression analysis between tumor and normal lung tissues identified a total of 14,637 DEGs, including 11,552 upregulated and 3085 downregulated genes (Figure 2A), indicating extensive transcriptional remodeling in LUAD. Subsequently, using 15 epithelial cell subpopulations defined by scRNA‐seq as a reference, BayesPrism was applied to deconvolute the TCGA bulk RNA‐seq samples, yielding a relative abundance spectrum of epithelial subpopulations (theta) for each sample and illustrating the compositional differences (Figure 2B).

**FIGURE 2:**
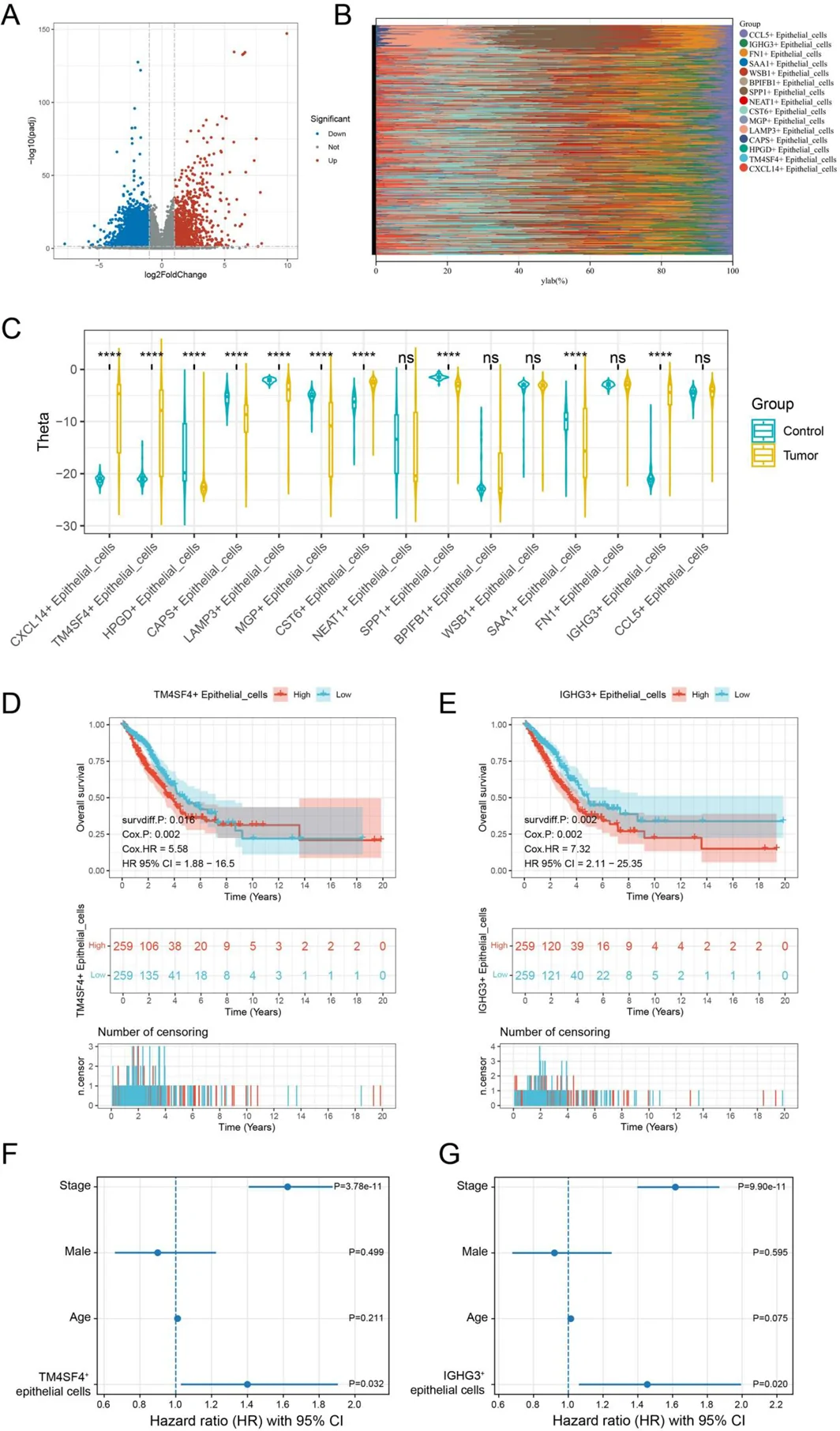
Deconvolution‐derived epithelial cell abundance reveals enrichment of the *TM4SF4* ^+^ subpopulation and predicts poor prognosis in lung adenocarcinoma. (A) Volcano plot of differentially expressed genes between TCGA‐LUAD tumor and normal tissues (red, upregulated; blue, downregulated; gray, not significant); (B) overview of BayesPrism deconvolution results showing the relative abundance (theta) of 15 scRNA‐seq‐defined epithelial subpopulations across TCGA samples; (C) tumor–normal comparisons of all 15 epithelial subpopulations, with theta plotted on a logarithmic scale; (D) KM OS analysis based on *TM4SF4* ^+^ epithelial cell abundance (theta), with patients dichotomized at the tumor‐sample median and univariable Cox results reported; (E) KM OS analysis for *IGHG3* ^+^ epithelial cells using the same stratification strategy, with univariable Cox results reported; (F) independent association between high *TM4SF4* ^+^ epithelial cell abundance and OS in a multivariable Cox model adjusting for age, gender, and pathological stage (HR and 95% CI); and (G) independent association between high *IGHG3* ^+^ epithelial cell abundance and OS in the corresponding multivariable Cox model (HR and 95% CI). OS, overall survival.

Comparison of theta values between tumor and normal tissues revealed significant changes in the abundance of multiple epithelial subpopulations (Figure 2C). Notably, *TM4SF4* ^+^ epithelial cells were significantly enriched in tumor tissues, suggesting that this subpopulation may play a role in LUAD progression. Further analysis of tumor samples at the patient level aggregated abundance data, divided patients into high‐ and low‐abundance groups based on the median abundance of *TM4SF4* ^+^ epithelial cells, and conducted OS analysis using the median cutoff (Table 1). In the median‐cutoff Cox analysis, high *TM4SF4* ^+^ abundance was associated with significantly shorter OS (HR = 1.43, 95% CI = 1.07–1.92, *p* = 0.016; Figure 2D). Similarly, a high abundance of *IGHG3* ^+^ epithelial cells was also associated with poorer OS (Figure 2E). KM results for the remaining 13 epithelial subpopulations are presented in the supplementary data (Supporting Information S1: Figure S4). The tumor–normal comparison statistics for all epithelial subtypes are summarized in Table 2.

**TABLE 1:** Sensitivity analysis of cutoff thresholds: robustness of the association between *TM4SF4* ^+^ and *IGHG3* ^+^ expression levels and overall survival under different cutoff strategies.

Marker	Cutoff method	Cutoff (theta)	Quantile (if applicable)	*N*	*N* (high/low)	HR (95% CI)	Cox *p*	Log‐rank *p*
*TM4SF4* ^+^ epithelial cells	Median	0.0063		503	252/251	1.43 (1.07–1.92)	0.016	0.016
*TM4SF4* ^+^ epithelial cells	Tertile (top vs. bottom)	0.0354	0.67	336	168/168	1.67 (1.17–2.37)	0.004	0.004
*TM4SF4* ^+^ epithelial cells	Optimal (log rank)	0.0620	0.74	503	131/372	1.83 (1.35–2.49)	9.76e−05	7.61e−05
*IGHG3* ^+^ epithelial cells	Median	0.0492		503	252/251	1.61 (1.20–2.17)	0.002	0.001
*IGHG3* ^+^ epithelial cells	Tertile (top vs. bottom)	0.0950	0.67	336	168/168	1.77 (1.22–2.56)	0.003	0.002
*IGHG3* ^+^ epithelial cells	Optimal (log rank)	0.0259	0.39	503	307/196	1.81 (1.31–2.51)	3.14e−04	2.55e−04

**TABLE 2:** Tumor–normal comparison of epithelial subtype abundances (theta) estimated by BayesPrism deconvolution in the TCGA‐LUAD cohort.

Cell subtype	Tumor theta, median (IQR)	Control theta, median (IQR)	Δmedian (T‐C) [95% CI]	Direction (tumor vs. control)	P (MW)	FDR (BH)	Log_2_FC (mean)
*IGHG3* ^+^ epithelial cells	0.0471 (0.00912–0.130)	4.71e‐07 (3.25e‐07–7.80e‐07)	0.0471 [0.0375, 0.0577]	↑	1.13e‐34	1.70e‐33	10.2
CST6^+^ epithelial cells	0.161 (0.0923–0.231)	0.0134 (0.00592–0.0263)	0.147 [0.136, 0.162]	↑	2.79e‐30	1.66e‐29	3.07
CXCL14^+^ epithelial cells	0.0394 (1.54e‐05‐0.133)	4.73e‐07 (3.12e‐07–5.85e‐07)	0.0394 [0.0278, 0.0547]	↑	3.32e‐30	1.66e‐29	17.2
SPP1^+^ epithelial cells	0.111 (0.0561–0.182)	0.354 (0.314–0.411)	−0.242 [−0.266, −0.221]	↓	4.26e‐29	1.60e‐28	−1.47
LAMP3^+^ epithelial cells	0.0690 (0.0157–0.155)	0.234 (0.206–0.298)	−0.165 [−0.197, −0.146]	↓	6.21e‐20	1.86e‐19	−1.19
CAPS^+^ epithelial cells	0.00250 (2.42e‐04‐0.00853)	0.0268 (0.0116–0.0426)	−0.0243 [−0.0295, −0.0126]	↓	1.08e‐19	2.70e‐19	−1.95
HPGD^+^ epithelial cells	1.63e‐07 (1.02e‐07‐2.70e‐07)	1.07e‐06 (3.67e‐07–7.42e‐04)	−9.10e‐07 [−4.07e‐06, −3.34e‐07]	↓	5.90e‐18	1.27e‐17	1.18
MGP^+^ epithelial cells	5.52e‐04 (6.59e‐07‐0.0121)	0.0290 (0.0175–0.0416)	−0.0285 [−0.0365, −0.0244]	↓	1.52e‐17	2.86e‐17	−1.46
*TM4SF4* ^+^ epithelial cells	0.00430 (6.69e‐07‐0.0636)	4.73e‐07 (3.63e‐07–6.60e‐07)	0.00430 [9.32e‐04, 0.00892]	↑	3.63e‐16	6.05e‐16	15.2
NEAT1^+^ epithelial cells	7.19e‐07 (3.45e‐07‐0.00329)	9.21e‐05 (9.92e‐07–0.00238)	−9.14e‐05 [−0.00104, −9.69e‐07]	↓	7.45e‐04	0.001	3.38
SAA1^+^ epithelial cells	1.93e‐05 (5.79e‐07‐0.00562)	0.00127 (3.38e‐04–0.00315)	−0.00125 [−0.00199, −8.15e‐04]	↓	9.10e‐04	0.001	1.57
CCL5^+^ epithelial cells	0.0545 (0.0264–0.0938)	0.0426 (0.0252–0.0607)	0.0119 [0.00243, 0.0218]	↑	0.015	0.019	0.555
WSB1^+^ epithelial cells	0.108 (0.0665–0.146)	0.121 (0.0755–0.150)	−0.0128 [−0.0349, 0.00392]	NS	0.275	0.318	0.0118
FN1^+^ epithelial cells	0.129 (0.0762–0.200)	0.128 (0.110–0.167)	0.00151 [−0.0186, 0.0121]	NS	0.627	0.672	0.137
BPIFB1^+^ epithelial cells	1.34e‐07 (7.83e‐08‐1.46e‐05)	1.36e‐07 (1.04e‐07–1.98e‐07)	−1.57e‐09 [−3.16e‐08, 2.53e‐08]	NS	0.974	0.974	4.32

*Note*: Theta denotes the relative abundance estimated by BayesPrism. Data are presented as median (IQR). Δmedian represents the median difference between tumor and control samples (tumor−control), with 95% CIs obtained by bootstrap resampling (2000 iterations). Group differences were assessed using two‐sided Mann–Whitney *U*‐tests, and false discovery rates (FDRs) were adjusted using the Benjamini–Hochberg procedure. Direction (↑/↓/NS) indicates significantly increased/decreased/not significant in tumors (FDR < 0.05).

To reduce the instability caused by cutoff selection, a sensitivity analysis was performed using multiple strategies, including median split, tertile comparison, and optimal cutpoint. Both *TM4SF4* ^+^ and *IGHG3* ^+^ epithelial cells retained consistent associations with poor prognosis across different approaches and remained significant (Table 1). Moreover, in multivariate Cox models adjusted for age, gender, and pathological stage, high abundance of *TM4SF4* ^+^ and *IGHG3* ^+^ epithelial cells was independently associated with OS (Figure 2F,G), supporting their roles as key epithelial subpopulations linked to poor prognosis in LUAD.

### 3.3 Association of *TM4SF4* ^+^ epithelial cell abundance with the immune microenvironment in LUAD

To evaluate the relationship between *TM4SF4* ^+^ epithelial cells and the immune microenvironment in LUAD, we aggregated the abundance (theta) of *TM4SF4* ^+^ epithelial cells obtained through BayesPrism deconvolution for each patient from TCGA‐LUAD tumor samples. Patients were divided into high‐ and low‐abundance groups using the median cutoff (theta = 0.00612) (*n* = 258 vs. 258). First, epithelial niche analysis suggested a significant remodeling of epithelial subpopulation composition in *TM4SF4* ^+^ high‐abundance tumors: the relative abundance of CXCL14^+^ and LAMP3^+^ epithelial cells was increased, while the relative abundance of SPP1^+^, FN1^+^, CCL5^+^, WSB1^+^, and CST6^+^ epithelial cells was decreased (Figure 3A). These findings indicate a specific epithelial state background associated with the *TM4SF4* ^+^ high‐abundance phenotype.

**FIGURE 3:**
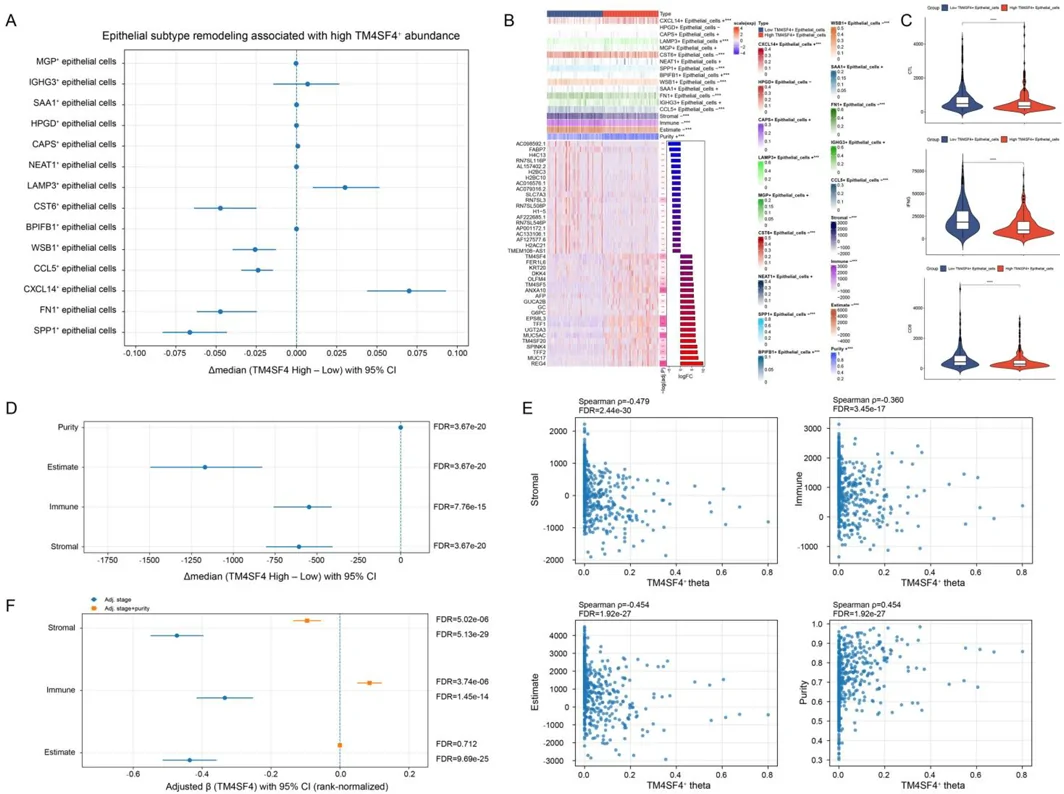
Association between *TM4SF4* ^+^ epithelial cell abundance and the immune microenvironment in lung adenocarcinoma. (A) Remodeling of the epithelial niche, showing differences in the abundance of other epithelial subpopulations between *TM4SF4* ^+^ high‐ and low‐abundance groups defined by the median cutoff; (B) differential gene expression patterns and ESTIMATE‐related scores between *TM4SF4* ^+^ high‐ and low‐abundance groups; (C) group comparisons of CTL, IFNG, and CD8; (D) unadjusted median differences in stromal score, immune score, ESTIMATE score, and tumor purity between the high‐ and low‐abundance groups, with 95% CIs; (E) correlation analysis treating *TM4SF4* ^+^ abundance (theta) as a continuous variable, evaluating its relationships with stromal score, immune score, ESTIMATE score, and purity; and (F) regression associations with stromal, immune, and ESTIMATE scores after adjustment for stage and for stage plus tumor purity.

At the immune microenvironment level, we further utilized the ESTIMATE algorithm to calculate the stromal score, immune score, and ESTIMATE score and to infer tumor purity (Purity) (Figure 3B). Results showed that compared with the low‐abundance group, the high‐abundance group exhibited significantly lower stromal score, immune score, and ESTIMATE score, whereas Purity was significantly higher (Figure 3D), collectively characterizing a “low immunity/stromal scores—high tumor purity” microenvironment. After adjustment for pathological stage, the associations with stromal, immune, and ESTIMATE scores remained significant (Figure 3F). To reduce dependence on the cutoff, we further evaluated *TM4SF4* ^+^ abundance as a continuous variable and found it to be significantly negatively correlated with stromal, immune, and ESTIMATE scores but positively correlated with Purity (Figure 3E), further supporting the aforementioned conclusions. Stratified and confounder‐adjusted analyses indicated that these associations remained significant after controlling for pathological stage; following further adjustment for Purity, *TM4SF4* ^+^ abundance maintained a robust independent negative correlation with stromal score. However, the residual association direction with immune score changed, implying that the “reduced immune score” is likely driven to a greater extent by tumor purity from a statistical perspective, while its association with reduced stromal components appears more stable (Figure 3F).

### 3.4 Correlation of *TM4SF4* ^+^ epithelial cell abundance with somatic mutations and drug sensitivity in LUAD

To further characterize the molecular features of *TM4SF4* ^+^ epithelial cell‐associated LUAD and explore its potential therapeutic implications, we systematically evaluated the somatic mutation profile and performed drug‐sensitivity prediction analysis using the TCGA‐LUAD cohort. An overview of the mutation profile revealed that *TP53* exhibited the highest mutation frequency, followed by TTN and MUC16 (Figure 4A,B). Comparing the high‐abundance and low‐abundance *TM4SF4* ^+^ epithelial cell groups, *TP53* and *KRAS* remained the most frequently mutated genes in both groups. However, their mutation composition showed differences before multiple‐testing correction: *TP53* mutations were more prevalent in the low‐abundance group, whereas *KRAS* mutations were more common in the high‐abundance group (Figure 4C).

**FIGURE 4:**
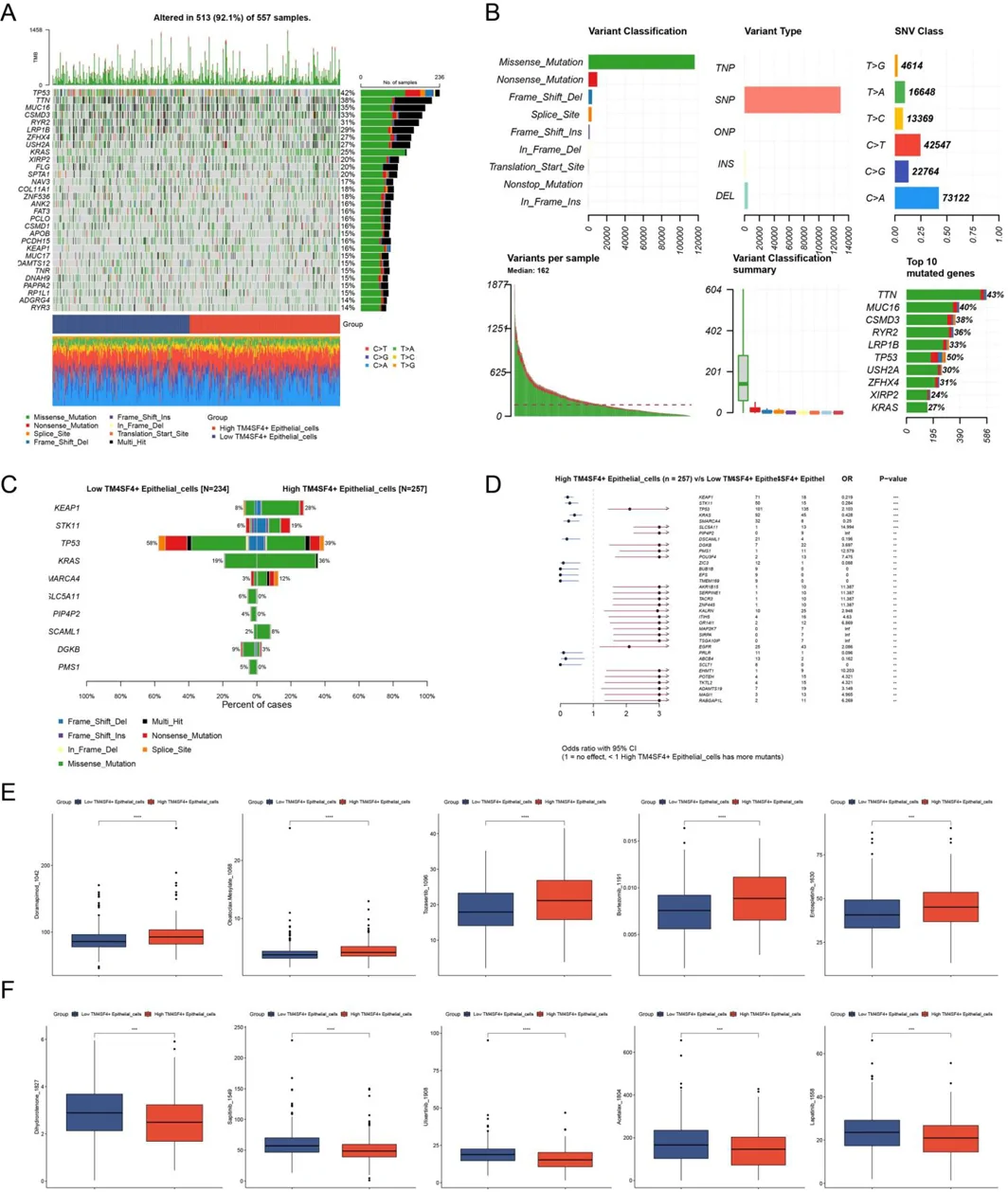
Somatic mutation landscape and drug‐sensitivity prediction associated with *TM4SF4* ^+^ epithelial cell abundance in lung adenocarcinoma. (A) Oncoplot depicting the somatic mutation landscape of the TCGA‐LUAD cohort; (B) summary statistics of somatic variants, including variant functional classifications, mutation types, single‐nucleotide variant substitution categories, distribution of mutations per sample, proportions of variant classes, and frequencies of top mutated genes; (C) stacked bar plot comparing the frequencies of recurrently mutated genes between *TM4SF4* ^+^ high‐ and low‐abundance groups; (D) forest plot summarizing genome‐wide differential mutation analysis between high‐ and low‐abundance groups based on per‐gene Fisher exact tests, with ORs, 95% CIs, raw *p* values, and BH‐FDR‐adjusted *p* values provided in Supporting Information S2: Table S2; (E) boxplots of predicted drug sensitivity for agents to which the *TM4SF4* ^+^ high‐abundance group is more responsive; and (F) boxplots of predicted drug resistance for agents to which the *TM4SF4* ^+^ high‐abundance group is less responsive. ORs, odds ratios.

At the mutational landscape level, *KRAS* mutations were more frequent and *TP53* mutations less frequent in tumors with high *TM4SF4* ^+^ epithelial cell abundance, but neither difference met the BH‐adjusted FDR threshold. More importantly, the high‐abundance group showed a notable enrichment of mutations in several key driver genes, including *KEAP1* and *STK11*, suggesting a co‐mutation landscape associated with metabolic stress, redox homeostasis, and inactivation of tumor suppression pathways (Figure 4D). The full differential mutation statistics are provided in Supporting Information S2: Table S2. Regarding therapeutic response, we further used oncoPredict to infer the drug response profiles of patients in the two groups. Compared with the low‐abundance group, the high‐abundance *TM4SF4* ^+^ group showed higher predicted response values to various drugs, including Doramapimod, Obatoclax Mesylate, Tozasertib, Bortezomib, and Entospletinib (Figure 4E), suggesting potential therapeutic sensitivity. Conversely, the high‐abundance group showed lower predicted response values to drugs such as Dihydrorotenone, Sapitinib, Ulixertinib, Acetalax, and Lapatinib (Figure 4F), suggesting relative resistance to specific pathway inhibitors.

### 3.5 Identification of *TM4SF4* ^+^ epithelium‐associated hub genes via WGCNA and multi‐evidence intersection, highlighting *MMP14* as a candidate target

To systematically identify key regulatory genes associated with *TM4SF4* ^+^ epithelial cell phenotypes, we constructed a WGCNA based on TCGA‐LUAD expression data. A total of 17 co‐expression modules were identified (Figure 5A), among which the MEtan module showed a significant positive correlation with *TM4SF4* ^+^ cell scores (Figure 5B), suggesting that this module may represent a *TM4SF4* ^+^‐associated co‐expression program. Subsequently, we cross‐referenced the genes in the MEtan module, DEGs from TCGA tumor–normal analysis, and marker genes of *TM4SF4* ^+^ epithelial cells identified via scRNA‐seq. This multi‐evidence approach yielded six candidate hub genes supported by consistent evidence: *LOXL1*, *DCBLD2*, *MMP14*, *MXRA5*, *VCAN*, and *PLAU* (Figure 5C). Notably, these six genes were significantly upregulated in tumor tissues compared with normal tissues in TCGA‐LUAD (Figure 5D).

**FIGURE 5:**
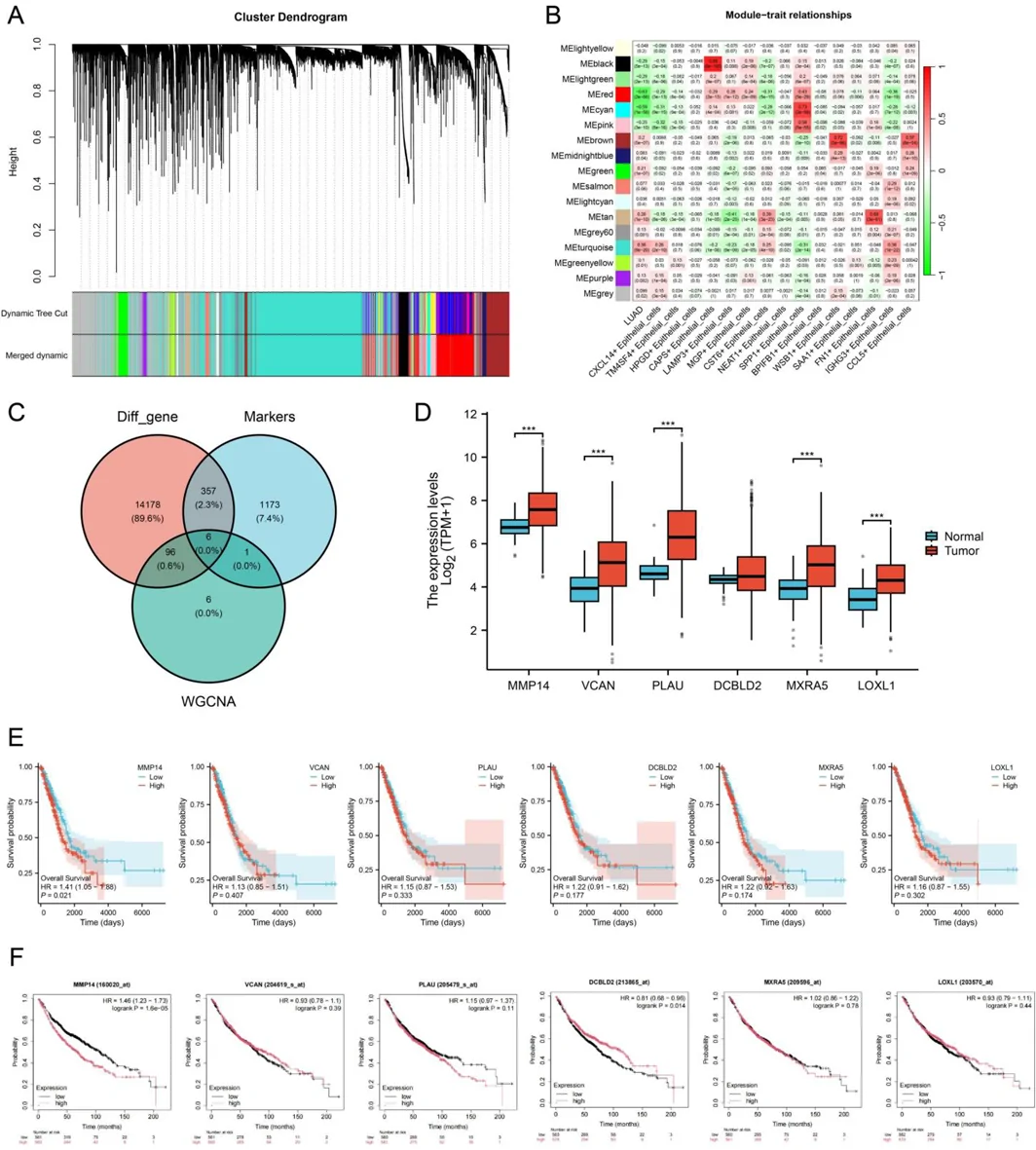
WGCNA and multi‐evidence integration identify *TM4SF4* ^+^‐associated hub genes and validate their expression and prognostic relevance. (A) Gene dendrogram from WGCNA module detection, showing hierarchical clustering and module assignment based on the TCGA‐LUAD co‐expression network; (B) module–trait correlation heatmap displaying correlation coefficients and significance levels between co‐expression modules and *TM4SF4* ^+^ cell scores, as well as related phenotypes; (C) venn diagram illustrating the overlap among MEtan module genes, TCGA tumor–normal differentially expressed genes, and *TM4SF4* ^+^ epithelial cell marker genes identified from scRNA‐seq; (D) boxplots comparing candidate gene expression between tumor and normal tissues; (E) KM curves evaluating the association between candidate gene expression and OS; and (F) KM survival analysis validating prognostic significance in an external cohort. WGCNA, weighted gene co‐expression network analysis.

To further evaluate the prognostic significance of these candidate genes, KM analysis in the TCGA cohort revealed variable survival associations across genes, with the high‐expression group of *MMP14* exhibiting worse OS (Figure 5E). A Cox proportional hazards model further confirmed the significant and independent association of *MMP14* with poor prognosis: univariate HR = 1.252 (95% CI: 1.102–1.423, *p* < 0.001) and multivariate‐adjusted HR = 1.378 (95% CI: 1.142–1.663, *p* < 0.001) (Table 3). In contrast, *DCBLD2* showed a weak correlation with OS in the univariate analysis but did not reach statistical significance in the multivariate model (*p* = 0.078). The remaining genes also lacked consistent independent prognostic relevance in multivariate analyses (Table 3). Validation in an external cohort (KM‐Plotter) showed results consistent with the aforementioned trend, with high *MMP14* expression indicating poorer prognosis (Figure 5F), providing independent supportive evidence for its prognostic value.

**TABLE 3:** Univariate and multivariate Cox analyses for candidate hub‐gene expression and overall survival.

Characteristics	Total (*N*)	Univariate analysis		Multivariate analysis	
		Hazard ratio (95% CI)	*p* value	Hazard ratio (95% CI)	*p*‐value
*LOXL1*	530	1.068 (0.924–1.235)	0.374	0.939 (0.781–1.129)	0.503
*DCBLD2*	530	1.130 (1.017–1.255)	0.023	1.138 (0.986–1.315)	0.078
*MMP14*	530	1.252 (1.102–1.423)	<0.001	1.378 (1.142–1.663)	<0.001
*MXRA5*	530	1.095 (0.987–1.216)	0.088	1.000 (0.810–1.236)	0.999
*VCAN*	530	1.084 (0.986–1.192)	0.097	0.985 (0.829–1.170)	0.863
*PLAU*	530	1.063 (0.976–1.157)	0.162	0.889 (0.772–1.023)	0.1

*Note*: Gene expression was dichotomized using the median cutoff. The multivariate model adjusted for age, gender, and pathological stage.

At the clinicopathological level, univariate logistic regression analysis demonstrated a positive correlation between high *MMP14* expression and tumor progression features, including advanced T stage, lymph node positivity, and higher overall pathological stage. However, no significant associations were observed with M stage, gender, age, or smoking history (Table 4). Collectively, the integration of WGCNA module association analysis with multi‐evidence intersection identified *MMP14* as a prioritized *TM4SF4* ^+^‐associated candidate hub gene. *MMP14* was significantly upregulated in tumors and correlated with poor prognosis and tumor progression features, thus providing a clear direction for future mechanistic studies and translational validation.

**TABLE 4:** Univariate logistic regression analysis of clinicopathologic factors associated with high *MMP14* expression in the TCGA‐LUAD cohort.

Characteristics	Total (*N*)	OR (95% CI)	*p*‐value
Pathologic T stage (T2&T3&T4 vs. T1)	536	1.486 (1.034–2.136)	0.032
Pathologic N stage (N1&N2&N3 vs. N0)	523	1.466 (1.016–2.116)	0.041
Pathologic M stage (M1 vs. M0)	390	0.825 (0.365–1.867)	0.645
Pathologic stage (Stage II&Stage III&Stage IV vs. Stage I)	531	1.523 (1.079–2.149)	0.017
Gender (male vs. female)	539	0.855 (0.610–1.200)	0.366
Age (>65 vs. ≤ 65)	520	0.955 (0.677–1.347)	0.792
Smoker (yes vs. no)	525	0.957 (0.590–1.552)	0.859

*Note*: High *MMP14* expression was defined using the median expression cutoff; odds ratios compare the high‐expression group with the corresponding reference category.

### 3.6 Functional enrichment analysis of *MMP14*‐associated transcriptional programs reveals ECM remodeling/migration‐invasion and immune‐related pathways

To elucidate the molecular functions and potential mechanisms associated with the upregulation of *MMP14*, single‐gene differential analysis was performed by dividing samples into high‐ and low‐*MMP14*‐expression groups, followed by the generation of ranked gene lists. Subsequently, GSEA was conducted to evaluate pathway enrichment, with significance determined by NES and FDR‐adjusted *q*‐values. The results demonstrated significant positive enrichment of multiple tumor‐related and immune dysfunction‐associated KEGG pathways in the high‐*MMP14*‐expression group, including representative pathways, such as bladder cancer, primary immunodeficiency, and NSCLC (Figure 6A). Additionally, the Hedgehog signaling pathway and the Notch signaling pathway exhibited significant enrichment (Figure 6A,C), suggesting a potential synergistic role of *MMP14*‐related transcriptional programs with oncogenic signaling axes. Taste transduction and systemic lupus erythematosus pathways had negative NES values in the *MMP14*‐high versus *MMP14*‐low comparison, consistent with relative enrichment in the low‐expression group (Figure 6B,D), indicating a stronger association of the low‐*MMP14*‐expression phenotype with specific immune‐related and non‐tumor metabolic sensory pathways.

**FIGURE 6:**
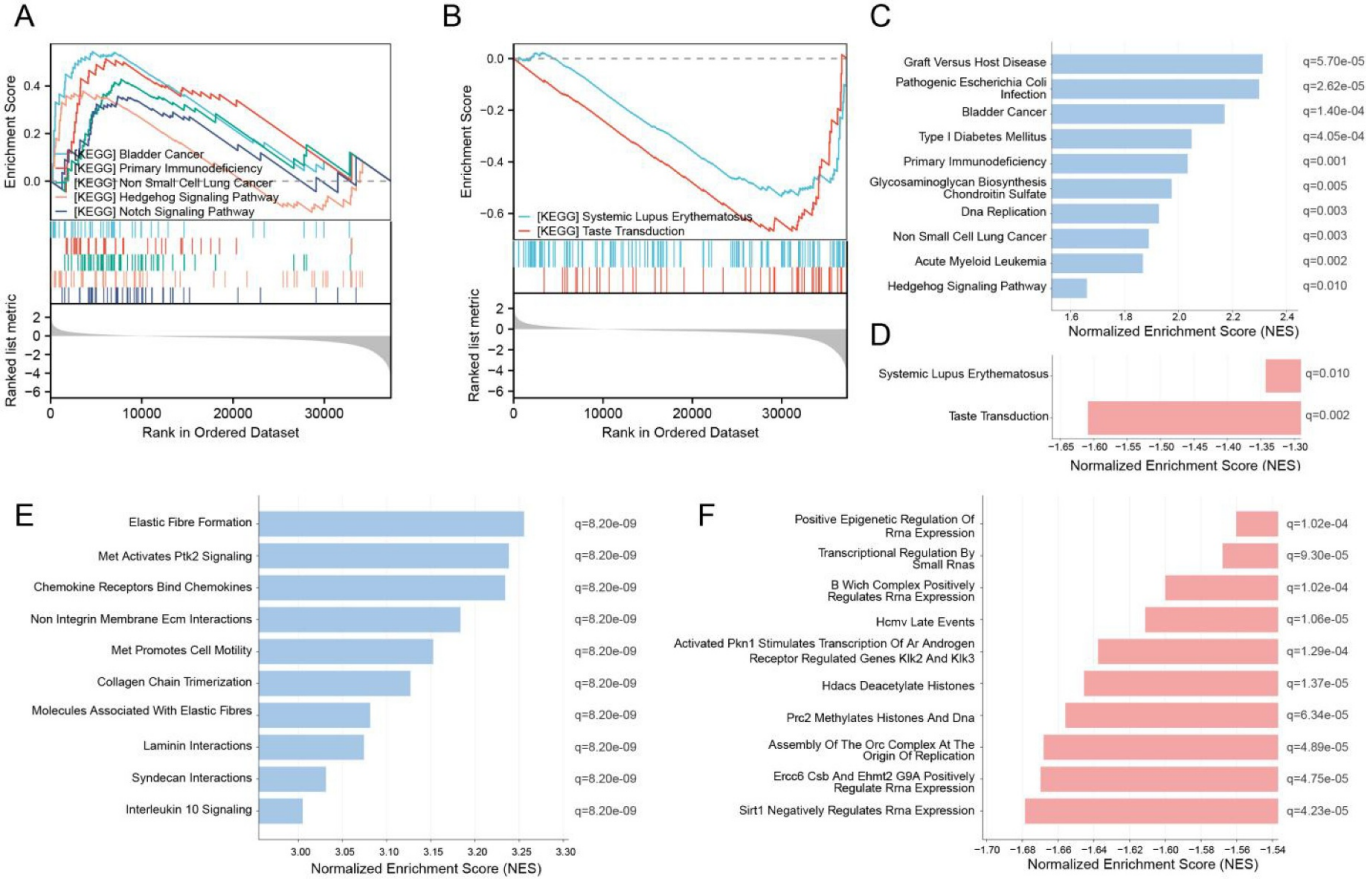
GSEA enrichment profiles and key pathway landscape associated with *MMP14* expression in lung adenocarcinoma. (A) GSEA enrichment curves for positively enriched KEGG pathways; (B) GSEA enrichment curves for negatively enriched KEGG pathways; (C) bar plot of NES for significantly enriched KEGG pathways in the *MMP14* high‐expression group; (D) bar plot of NES values for significantly enriched KEGG pathways in the *MMP14* low‐expression group; (E) bar plot of NES values for significantly enriched Reactome pathways in the *MMP14* high‐expression group; and (F) bar plot of NES values for significantly enriched Reactome pathways in the *MMP14* low‐expression group. GSEA, gene set enrichment analysis; KEGG, Kyoto Encyclopedia of Genes and Genomes; NES, normalized enrichment score.

To further enhance the mechanistic understanding of the “invasive phenotype,” we focused on Reactome pathways significantly enriched in the high‐*MMP14*‐expression group that are associated with ECM remodeling, cell–ECM interactions, and migratory activities. Notable pathways included elastic fiber formation, non‐integrin membrane ECM interactions, Met promotes cell motility, and collagen chain trimerization (Figure 6E), collectively highlighting the close link between *MMP14* upregulation and enhanced ECM remodeling and migration–invasion processes. In contrast, the low‐*MMP14*‐expression group exhibited significant enrichment in Reactome pathways predominantly related to transcriptional and epigenetic regulatory biological processes, further underscoring the systematic functional pathway differences between high‐ and low‐expression phenotypes (Figure 6F). Synthesizing the enrichment results from both KEGG and Reactome analyses, the *MMP14* high‐expression‐associated programs reveal a consistent functional directionality of “ECM remodeling—migration‐invasion—immune pathway alterations,” providing mechanistic insights into its role in the *TM4SF4* ^+^‐related LUAD phenotype.

### 3.7 Spatial transcriptomics validates a structured, spatially heterogeneous distribution of *MMP14* within LUAD tissue

Using spatial transcriptomics data from GSM5702474 and GSM5388415, *MMP14* exhibits pronounced spatial heterogeneity in LUAD and normal distal lung tissues. In the GSM5702474 section, *MMP14* signals form localized high‐expression “hotspots” with an approximately elliptical clustering pattern, suggesting that its expression may be linked to specific microenvironmental niches such as the tumor core or the invasive front (Figure 7A). In contrast, the normal distal lung section GSM5388415 shows a more diffuse *MMP14* signal with an elongated spatial distribution (Figure 7B). Collectively, these sections show heterogeneous *MMP14* expression in LUAD and normal distal lung tissues.

**FIGURE 7:**
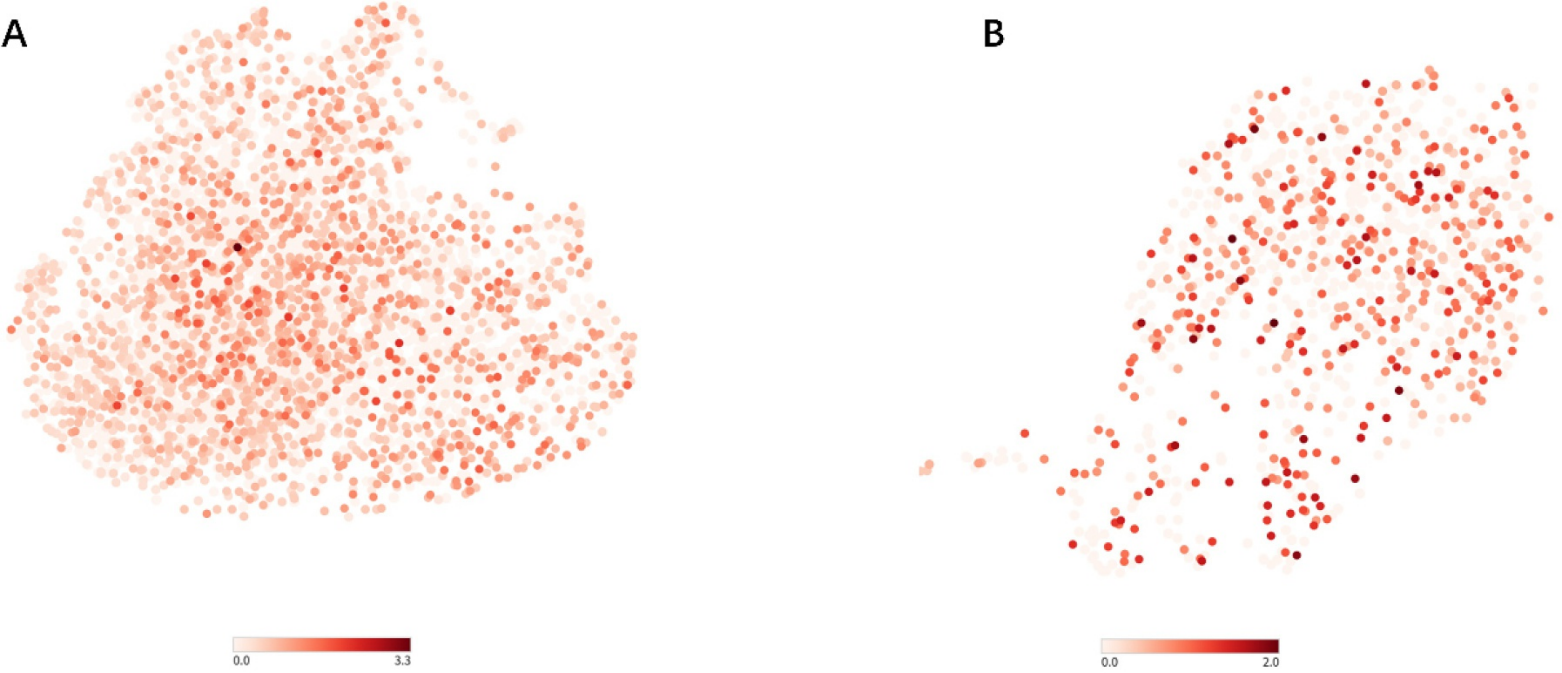
Spatially heterogeneous distribution of *MMP14* in spatial transcriptomic sections. (A) Spatial expression pattern of *MMP14* in the spatial transcriptomics dataset GSM5702474 and (B) Spatial expression of *MMP14* in the normal distal lung section GSM5388415. Color intensity from light to dark indicates increasing expression levels.

### 3.8 Cellular‐ and tissue‐level validation confirms marked upregulation of *MMP14* in LUAD

To validate *MMP14*, the hub gene prioritized by our bioinformatic analyses, we assessed its expression at both the cellular and clinical tissue levels. RT‐qPCR showed that *MMP14* mRNA expression is significantly higher in the LUAD cell line A549 than in the normal bronchial epithelial cell line BEAS‐2B (Figure 8A), indicating upregulation in tumor epithelial cells. Consistently, semiquantitative IHC of paired clinical samples demonstrates substantially elevated *MMP14* protein expression in tumor tissue relative to adjacent non‐tumor tissue (Figure 8B). Representative H&E and IHC sections further reveal stronger brown chromogenic staining in tumor regions, with focal areas of signal enrichment (Figure 8C). Together, these results show concordant *MMP14* upregulation at both the mRNA and protein levels in LUAD, supporting its role as a functional hub associated with the *TM4SF4* ^+^ phenotype and highlighting its potential as a translational target.

**FIGURE 8:**
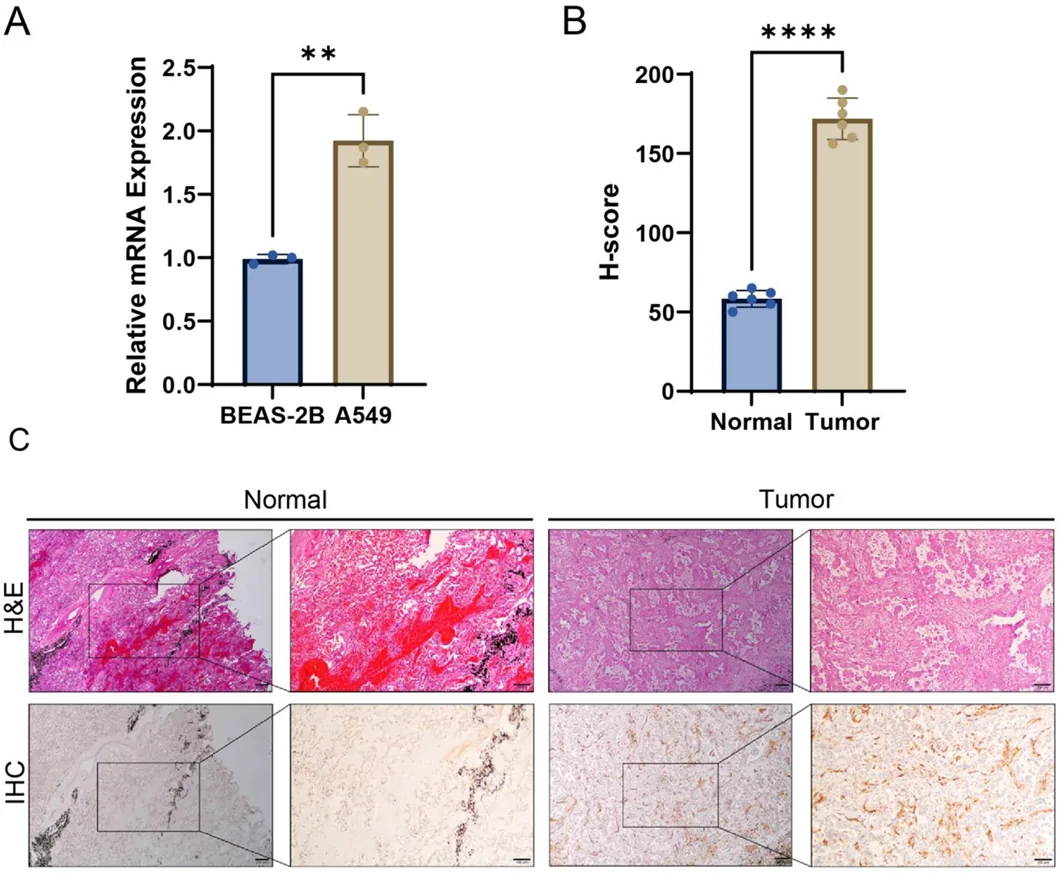
Validation of *MMP14* expression in lung adenocarcinoma cell lines and clinical tissues. (A) Bar plot showing relative expression measured by RT‐qPCR; (B) bar plot of semiquantitative IHC H‐scores; and (C) representative histological and IHC images of adjacent normal and tumor tissues, including H&E staining and *MMP14* IHC staining (scale bar = 40 μm) with corresponding magnified views (scale bar = 20 μm). **p < 0.01 and ****p < 0.0001.

## 4 DISCUSSION

Lung cancer remains one of the leading causes of cancer‐related mortality worldwide, with GLOBOCAN 2022 data indicating its persistent high burden of death.^1^ Although targeted therapy and immunotherapy have significantly improved the prognosis of certain patients, acquired resistance and tumor intrinsic/extrinsic heterogeneity continue to represent major challenges limiting the durability of therapeutic efficacy.^5^, ^9^, ^19^ The organizational complexity and cellular diversity of LUAD result in highly variable responses to identical treatment regimens among different patients, thereby fundamentally constraining the implementation of precision therapy. Against this backdrop, the present study integrates scRNA‐seq, GWAS, and bulk transcriptomic data to systematically analyze the epithelial heterogeneity of LUAD and, for the first time, identifies *TM4SF4* ^+^ epithelial cell subsets as the core cellular state of LUAD based on genetic susceptibility. This subset not only ranked highest in GWAS trait‐association scores but was also significantly associated with an immunosuppressive TME, specific mutational profiles, and poor prognosis. Compared with conventional approaches relying solely on expression‐based clustering for subgroup identification, this study establishes an integrative framework of “genetic risk‐cellular state‐clinical outcome,” enhancing the causal interpretability and translational applicability of key subset identification. This cross‐scale analytic paradigm provides novel theoretical insights into LUAD molecular subtyping while offering cellular‐level support for precision medicine strategies.

*TM4SF4* has been previously reported to be associated with migration, invasion, and epithelial–mesenchymal transition (EMT) in epithelial tumors, such as hepatocellular carcinoma and colorectal cancer,^20^, ^21^ primarily regarded as a “metastatic marker.” In contrast, this study identifies *TM4SF4* as a core marker of specific epithelial cell subpopulations and uncovers the regulatory role of these subpopulations within the TME, signifying a paradigm shift from the concept of “a single molecular function” to “a regulator of cellular states.” This *TM4SF4* ^+^ cell subpopulation exhibits distinct transcriptional features, including significantly upregulated expression of matrix metalloproteinases (e.g., *MMP14*), suggesting its involvement in ECM remodeling and invasive progression.^22^ Unlike previous studies that focused solely on single‐gene expression, this research emphasizes the role of *TM4SF4* ^+^ cells as functional units in modulating the tumor niche. Furthermore, we integrated GWAS signals into single‐cell resolution analyses, marking the first application of this approach in LUAD research and providing genetic support for the identification of these subpopulations. The findings not only align with previous reports of *TM4SF4*'s pro‐tumorigenic functions but also expand its biological implications at a higher conceptual level.

The abundance of the *TM4SF4* ^+^ subpopulation is significantly negatively correlated with OS, indicating its independent prognostic value. Given that PD‐L1 detection is influenced by factors such as sample acquisition, tumor heterogeneity, and scoring reproducibility, its predictive capacity remains limited.^23^ Therefore, novel cellular state markers are more likely to complement PD‐L1 in refining risk stratification and predictive systems for therapeutic efficacy.^24^ Based on integrated data analysis, we propose that its adverse prognostic effects may be mediated through two major pathways: first, by linking MMP14‐associated ECM degradation programs with invasion and metastatic potential and, second, by fostering an immunosuppressive TME, thereby promoting immune evasion. Specifically, high‐abundance *TM4SF4* ^+^ samples exhibit reduced IFNG signaling, decreased CD8^+^ T cell infiltration, and lower immune and stromal scores. This phenotype closely mirrors known characteristics of ICI resistance,^19^, ^25^ suggesting that this subpopulation may contribute to the establishment of an “immune‐privileged” TME. Additionally, potential regulatory alterations in the chemokine network may play a key role in modulating immune cell migration and infiltration. These findings underscore the importance of incorporating analyses of subpopulation composition into the design of immunotherapy strategies.

At the mutational landscape level, *KRAS* mutations are significantly enriched in patients with high *TM4SF4* expression, whereas the proportion of *TP53* mutations is reduced. This pattern differs from the common *KRAS*‐*TP53* co‐mutation structure observed in certain LUAD cases, suggesting that this subgroup could represent an independent evolutionary pathway specific to a distinct molecular subtype. Drug‐sensitivity analysis further indicates that this subgroup is potentially more susceptible to proteasome inhibitors, such as Bortezomib, while showing a resistance trend to EGFR inhibitors. This discrepancy may be attributed to the sustained activation of *KRAS*‐driven signaling pathways and the subgroup‐specific transcriptional regulatory networks. These findings suggest that the composition of tumor cell subgroups may directly impact the response to targeted therapies and should be integrated into therapeutic decision‐making. Although these observations require validation through in vitro experiments, they provide a theoretical foundation for precision medicine strategies based on cell state.

As a core effector molecule of the *TM4SF4* ^+^ subset, *MMP14* has been validated at multiple levels for its association with malignant characteristics. Beyond its direct involvement in ECM hydrolysis, the MMP‐related proteolytic network indirectly influences tumor cell plasticity and TME communication by altering ECM mechanics and receptor–ligand availability.^18^ Its expression profile partially overlaps with EMT markers, indicating its critical role in invasion and metastasis. Unlike previous studies that focused solely on *MMP14*, this study situates it within a specific cell subset, elucidating the coupling between its function and cellular states. Spatial transcriptomics further reveals a heterogeneous spatial distribution of *MMP14* in LUAD and normal distal lung sections. Therefore, the *TM4SF4*/*MMP14* axis may represent a key regulatory pathway in LUAD progression, offering a robust foothold for the development of targeted therapies.

Despite using an integrative multi‐omics strategy, this study still has several limitations. First, the single‐cell atlas was derived from GSE196303, which includes three LUAD tumor samples and three matched adjacent non‐tumor samples. Although Harmony integration and cluster‐stability assessment were used to reduce technical and patient‐specific effects, independent LUAD scRNA‐seq cohorts are still needed to confirm the reproducibility and marker structure of the *TM4SF4* ^+^ epithelial cluster. Second, copy‐number alteration or inferCNV analysis across epithelial clusters was not performed; therefore, malignant‐state interpretation was based on transcriptomic, deconvolution, mutation, spatial, RT‐qPCR, and IHC evidence rather than direct copy‐number inference. Third, the cellular origin and evolutionary trajectory of *TM4SF4* ^+^ epithelial cells remain unresolved and require future pseudotime, spatially informed trajectory, or lineage‐tracing analyses. Fourth, the scPagwas analysis used IEU OpenGWAS summary statistics with 1002 cases and 462,008 controls, and this case–control imbalance may increase the possibility that trait‐relevance scores reflect a limited number of strong loci rather than a broad polygenic architecture; accordingly, the scPagwas results should be interpreted as prioritization evidence rather than definitive causal proof. Fifth, the results of bulk deconvolution analysis rely on algorithmic assumptions and may be affected by sample complexity. Sixth, this study primarily utilizes retrospective data from publicly available databases, lacking prospective cohort validation. Seventh, the regulatory relationship between *TM4SF4* and *MMP14* has not yet been mechanistically validated using gene‐editing, knockdown/overexpression, organoid, or in vivo models. Eighth, drug‐sensitivity predictions are based on in silico models and require further validation in vitro or in patient‐derived xenograft models. Moreover, interactions between other cell types within the TME, such as dendritic cells and myeloid‐derived suppressor cells, and *TM4SF4* ^+^ subpopulations remain poorly elucidated. These limitations highlight the need for future studies to advance both mechanistic insights and clinical validation.

In conclusion, this study systematically identified and characterized a *TM4SF4* ^+^ epithelial cell subset in LUAD, revealing its association with genetic susceptibility, the immunosuppressive TME, poorer prognosis, and specific mutational backgrounds, while prioritizing *MMP14* as a closely related candidate hub gene. These findings enhance the understanding of LUAD epithelial heterogeneity and suggest that the *TM4SF4*/*MMP14*‐associated program may provide a theoretical basis for future therapeutic exploration. Scientifically, this study established an analytical paradigm driven by GWAS signals for identifying cell subsets at the single‐cell level, providing a model for integrative multi‐omics studies of tumor heterogeneity. Clinically, the *TM4SF4* ^+^ subset and its expression profile may serve as candidate biomarkers and risk‐stratification indices. Future efforts should focus on mechanistic studies, cross‐center clinical validation, and functional evaluation of *TM4SF4* ^+^ cell subsets to clarify their translational potential.

## 5 CONCLUSION

This study integrates scRNA‐seq, scPagwas, TCGA deconvolution, and multi‐omics validation to systematically characterize epithelial cell heterogeneity in LUAD and prioritize disease‐associated epithelial cell states. We identified 15 epithelial cell subpopulations, among which *TM4SF4* ^+^ epithelial cells demonstrated the highest TRS in scPagwas and were significantly associated with genetic risks of LUAD. In TCGA‐LUAD, this subpopulation was significantly enriched in tumor tissues, with high abundance stably predicting poorer OS and showing potential independent prognostic value. Tumors with high *TM4SF4* ^+^ abundance displayed a microenvironment characterized by “low immune/stromal scores—high tumor purity,” accompanied by specific epithelial niche remodeling and enrichment of *KRAS*/*KEAP1*/*STK11* driver mutations, along with distinct drug‐sensitivity prediction profiles. Furthermore, WGCNA and converging multi‐evidence analysis identified *MMP14* as a candidate hub gene associated with *TM4SF4* ^+^ cells. Functional enrichment analyses suggested its involvement in ECM remodeling and migration/invasion pathways. Spatial transcriptomics revealed a structured heterogeneous distribution of *MMP14*, with its mRNA and protein levels significantly upregulated in both cell lines and clinical tissues. In summary, *TM4SF4* ^+^ epithelial cells and the associated *MMP14* program may serve as stratification markers and candidate therapeutic targets in LUAD, providing a basis for future risk‐assessment and precision‐therapy studies.

## AUTHOR CONTRIBUTIONS

Kai Fu and Jingbo Li conceived and designed the study. Jiahua Liu, Guangxuan Liu, Kaiyu Wang, Qi Yan, Tianhao Sun, and Shuo Chen performed the experiments. Jingbo Li and Jiahua Liu analyzed the data. Jiahua Liu and Kai Fu wrote the manuscript. All authors reviewed and approved the final version of the manuscript.

## CONFLICT OF INTEREST STATEMENT

The authors declare no conflicts of interest.

## ETHICS STATEMENT

This study was approved by the Clinical Ethics Committee of The First Affiliated Hospital of Harbin Medical University.

## CONSENT

Not applicable.

## Supporting information

Supporting Information S1

Supporting Information S2

## Data Availability

All data generated or analyzed during this study are included in this article and/or its supplementary material files. Further enquiries can be directed to the corresponding author.
