# Supplementary material for: Multi‐omics analysis highlights TM4SF4 + epithelial cells and the MMP14‐associated signaling program in lung adenocarcinoma pathogenesis and microenvironment remodeling
Source: J Cell Commun Signal. 2026 Nov 13;20(4):e70114. doi: 10.1002/ccs3.70114 (PMC13760549; doi:10.1002/ccs3.70114)
Supplement: Supplementary file 1 [file CCS3-20-e70114-s001.docx]

**Figure S1. Schematic Overview of the Study Workflow.**

**Figure S2. Clustering Resolution and Stability Assessment for the scRNA-seq Atlas.**

Note: This supplementary figure corresponds to the former Figure 1B and shows the clustree-based clustering dendrogram used to evaluate clustering behavior across a numerical resolution grid before final cell-type annotation. The tested resolutions were 0.01, 0.05, 0.1, 0.2, 0.3, 0.5, 0.8, 1, 1.2, 1.5, 2, 2.5, and 3; the final whole-atlas clustering used resolution 1 based on the balance between cluster granularity and dendrogram continuity.

**Figure S3. Marker-Gene Heatmap Across the 15 Epithelial Subpopulations.**

Note: This supplementary figure corresponds to the former Figure 1G and presents representative marker-gene expression patterns across epithelial subpopulations. Marker readability was checked in the revised exported image; top marker-gene statistics for each epithelial cluster are provided in Supplementary Table S1.

**Figure S4. Association Between the Abundance of Other Epithelial Subpopulations and OS in the TCGA-LUAD Cohort.**

Note: Relative abundances of epithelial subpopulations are estimated using BayesPrism. Patients are stratified into high- and low-abundance groups according to a predefined cutoff, and KM survival curves are generated. Each panel reports the log-rank p-value (survdiff.p), Cox regression p-value (Cox.p), HR with 95% CI, and includes the number-at-risk table and censoring distribution. (A) BPIFB1+ epithelial cells; (B) CAPS+ epithelial cells; (C) CCL5+ epithelial cells; (D) CST6+ epithelial cells; (E) CXCL14+ epithelial cells; (F) FN1+ epithelial cells; (G) HPGD+ epithelial cells; (H) LAMP3+ epithelial cells; (I) MGP+ epithelial cells; (J) NEAT1+ epithelial cells; (K) SAA1+ epithelial cells; (L) SPP1+ epithelial cells; (M) WSB1+ epithelial cells.
